# Development of a risk score for early saphenous vein graft failure: An individual patient data meta-analysis

**DOI:** 10.1016/j.jtcvs.2019.07.086

**Published:** 2020-07

**Authors:** Alexios S. Antonopoulos, Ayodele Odutayo, Evangelos K. Oikonomou, Marialena Trivella, Mario Petrou, Gary S. Collins, Charalambos Antoniades, SAFINOUS-CABG (Saphenous Vein Graft Failure—An Outcomes Study in Coronary Artery Bypass Grafting) group, Ioannis Akoumianakis, Keith M. Channon, Laura Herdman, Marios Margaritis, Stefan Neubauer, Sheena Thomas, Stephen Fremes, Reena Karkhanis, Jeffrey Rade, Toshihiro Fukui, Hidefumi Nishida, Shuichiro Takanashi, Ho Young Hwang, Ki-Bong Kim, Luigi Mannacio, Vito Mannacio, Jota Nakano, Louis Perrault, Attila Kardos, Hitoshi Okabayashi, Dimitris Tousoulis, Andrew Kelion, Nik Sabharwal, George Krasopoulos, Rana Sayeed, David Taggart

**Affiliations:** a Division of Cardiovascular Medicine, Radcliffe Department of Medicine, University of Oxford, Oxford, United Kingdom; b Centre for Statistics in Medicine, University of Oxford, Oxford, United Kingdom; c Department of Cardiac Surgery, John Radcliffe Hospital, Oxford University Hospitals NHS Trust, Oxford, United Kingdom

**Keywords:** coronary artery bypass grafting, saphenous vein graft, individual patient meta-analysis, patency, prediction model, CABG, Coronary artery bypass grafting, CI, confidence interval, IPD, individual patient data, LIMA, left internal mammary artery, MLMI, multilevel multiple imputation, SAFINOUS-CABG, Saphenous Vein Graft Failure---An Outcomes Study in Coronary Artery Bypass Grafting, SVG, saphenous vein graft

## Abstract

**Objectives:**

Early saphenous vein graft (SVG) occlusion is typically attributed to technical factors. We aimed at exploring clinical, anatomical, and operative factors associated with the risk of early SVG occlusion (within 12 months postsurgery).

**Methods:**

Published literature in MEDLINE was searched for studies reporting the incidence of early SVG occlusion. Individual patient data (IPD) on early SVG occlusion were used from the SAFINOUS-CABG Consortium. A derivation (n = 1492 patients) and validation (n = 372 patients) cohort were used for model training (with 10-fold cross-validation) and external validation respectively.

**Results:**

In aggregate data meta-analysis (48 studies, 41,530 SVGs) the pooled estimate for early SVG occlusion was 11%. The developed IPD model for early SVG occlusion, which included clinical, anatomical, and operative characteristics (age, sex, dyslipidemia, diabetes mellitus, smoking, serum creatinine, endoscopic vein harvesting, use of complex grafts, grafted target vessel, and number of SVGs), had good performance in the derivation (c-index = 0.744; 95% confidence interval [CI], 0.701-0.774) and validation cohort (c-index = 0.734; 95% CI, 0.659-0.809). Based on this model. we constructed a simplified 12-variable risk score system (SAFINOUS score) with good performance for early SVG occlusion (c-index = 0.700, 95% CI, 0.684-0.716).

**Conclusions:**

From a large international IPD collaboration, we developed a novel risk score to assess the individualized risk for early SVG occlusion. The SAFINOUS risk score could be used to identify patients that are more likely to benefit from aggressive treatment strategies.

## Graphical abstract

Summary of the study design and the main findings of each arm. In Arm A, we performed a meta-analysis of the published literature to estimate the incidence of early SVG occlusion post-CABG. In Arm B, using IPD we developed a prediction model and the SAFINOUS score for the estimation of early SVG occlusion risk. *CABG*, Coronary artery bypass grafting; *SVG*, saphenous vein graft; *IPD*, individual patient data; *CV*, coefficient of variation; *AUC*, Area under curve; *CI*, confidence interval; *Glu*, glucose; *CKD*, chronic kidney disease.

**Figure:**
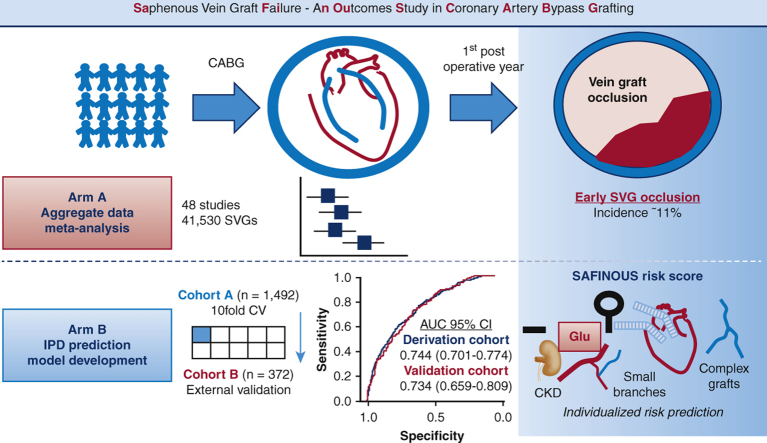

**Figure:**
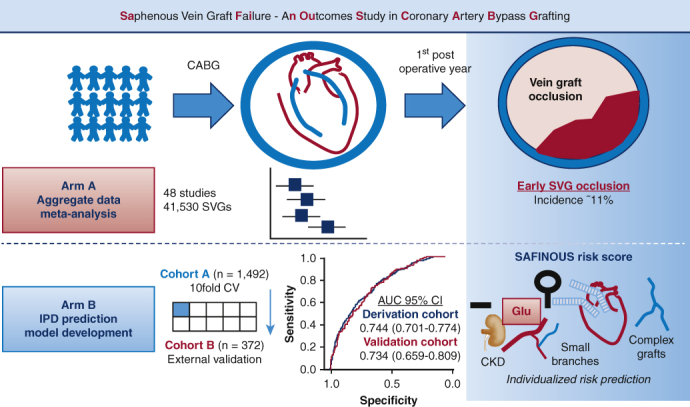
Summary of the study design and the main findings of each arm.

**Central Message**

A novel risk score (SAFINOUS score) estimates the individualized risk for early vein graft failure based on clinical, anatomical, and operative factors.

**Perspective**

The risk factors for early saphenous vein graft occlusion remain poorly understood as well as the optimal management of patients postoperatively. The developed SAFINOUS score could contribute to surgery planning or the guidance of treatment strategies postoperatively.

See Commentaries on pages 128 and 130.

Coronary artery bypass grafting (CABG) surgery is a widely used revascularization strategy for complex multivessel coronary artery disease that improves prognosis and patients' quality of life.^1^ Although left internal mammary artery (LIMA) graft is the gold standard for left anterior descending revascularization, the saphenous vein is the most widely used conduit for CABG.^2^ However, saphenous vein grafts (SVGs) have a greater rate of both early and late occlusion compared with arterial grafts.^3^

Late SVG occlusion is attributed to atherosclerosis, whereas early SVG occlusion (<12 months) is caused by thrombosis and/or intimal hyperplasia.^2^ Traditionally, early SVG occlusion is attributed to technical factors, but it could be affected by patient characteristics and/or operative factors as well. Although the long-term patency of SVGs has been extensively studied in several clinical studies,^4^ early SVG occlusion remains unclear. Surprisingly, there is a large discrepancy in the reported rates of SVG occlusion early post-CABG at 12 months.^5^ Previous reports suggest that female sex,^6^ diabetes mellitus,^7^ or off-pump surgery^8^ may affect graft patency; however, there is no comprehensive prediction model for early SVG occlusion.

A prediction model for early SVG occlusion could guide the deployment of effective prevention strategies. For example, dual antiplatelet treatment may improve early SVG patency,^9^ but the lack of cardiovascular mortality benefit^10^ and the risk of bleeding complications preclude its use in all patients with CABG.^11^ An individualized risk score model for early SVG occlusion could address this unmet need, eg, by personalized early postoperative administration of dual antiplatelet treatment or aggressive lipid-lowering treatment to high-risk patients^12^ to improve clinical outcomes, quality of life, and related health care costs post-CABG. Since the risk factors for early SVG occlusion remain unknown, we used an individual patient data (IPD) meta-analysis to develop a predictive model for early SVG occlusion.

## Material and Methods

### Study Design and Objectives

**Figure 1:**
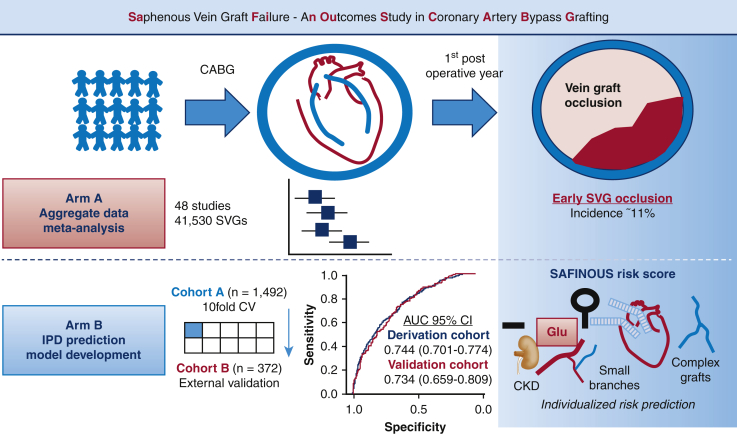
Summary of the study design and the main findings of each arm. *CABG*, Coronary artery bypass grafting; *SVG*, saphenous vein graft; *IPD*, individual patient data; *CV*, coefficient of variation; *AUC*, area under the curve; *CI*, confidence interval; *Glu*, glucose; *CKD*, chronic kidney disease.

The design of the study is summarized in the Figure 1.

#### Arm A: Systematic review of the literature for early SVG occlusion

Published literature in MEDLINE was systematically searched for studies describing the incidence of SVG occlusion during the first year post-CABG to provide an accurate estimation of early SVG occlusion.

#### Arm B: IPD meta-analysis and prediction model development

The SAFINOUS-CABG Consortium (Saphenous Vein Graft Failure---An Outcomes Study in Coronary Artery Bypass Grafting) is an international collaboration between cardiothoracic centers that has been formed with the aim to share IPD. By using IPD, a prediction model for early SVG occlusion was developed and internally validated in a derivation cohort (n = 1492 patients), which was subsequently tested in the validation cohort (n = 372). The aim was to construct a risk score for the individualized prediction of early graft occlusion that could be used as a clinical tool.

### Literature Search and Study Eligibility

#### Eligibility criteria

Eligibility criteria for including studies using the PICOS approach were as follows. Types of studies: clinical cohorts, registries, or randomized clinical trials; types of participants: patients with established coronary disease; types of interventions: CABG operation; types of outcome measures: the primary outcome was SVG occlusion within the first year post-CABG (assessed either by invasive angiography or by computed tomography angiography). No secondary outcomes were assessed.

#### Search methods for identification of studies

**Figure E1:**
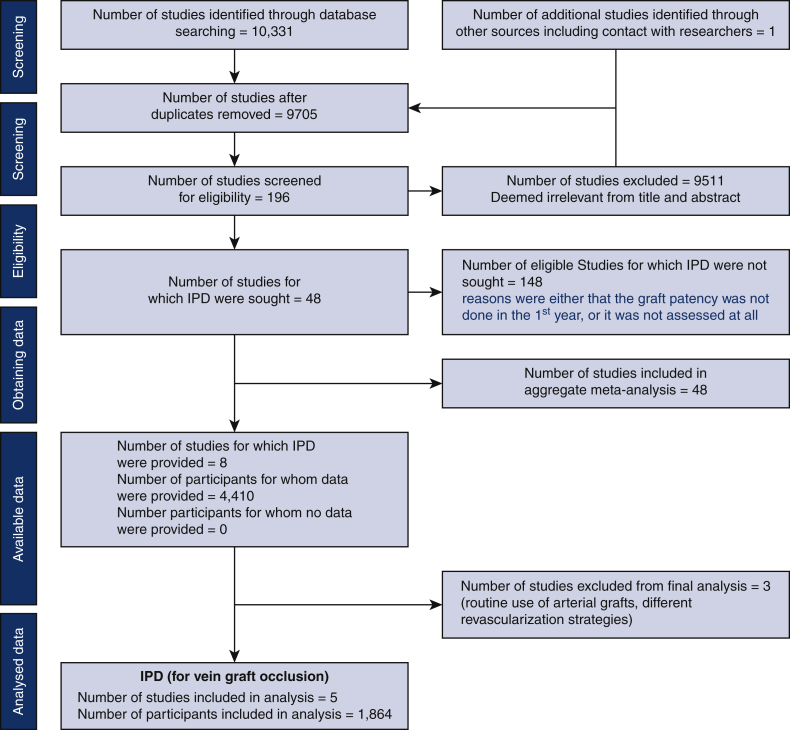
PRISMA-IPD flow chart for the study. *IPD*, Individual patient data.

Published literature was assessed by 2 independent reviewers (A.A. and E.O.) and then identified studies were further screened by a third independent reviewer (M.H.T.). Eligible studies were drawn from a systematic review of the English literature in Medline database from January 1970 until August 2017. The following medical subject headings (Medical Subject Headings terms) were used: “saphenous vein,” “coronary artery bypass,” “graft, occlusion,” “vascular patency,” and “coronary artery disease.” Studies were deemed eligible for inclusion if they were full-length publications in peer-reviewed journals, reporting on SVG patency rates within the first year after CABG either by invasive coronary angiography or computed tomography angiography. Graft occlusion was defined as a 100% stenosis of at least 1 SVG. From a total of 10,332 hits, 48 eligible studies were identified. The Preferred Reporting Items for Systematic Reviews and Meta-Analyses–IPD flow-chart for the study is presented in the Figure E1. The TRIPOD checklist^13^ is also included in the Online Data Supplement.

#### Measures of treatment effect

Studies may report the incidence of graft failure either as percent of patients enrolled, or as percent of grafts used. The primary outcome was expressed as the percent proportion of SVG occlusion per grafts used, since this information could be more reliably extracted from the aggregate published data.

### Developing a Predictive Model for Early SVG Occlusion Using IPD

To develop a predictive model for early SVG occlusion, shared IPD was used from the SAFINOUS-CABG Consortium, an international collaboration between cardiothoracic centers with IPD for early SVG patency in patients undergoing CABG. Authors of eligible articles were contacted per e-mail and/or per post with a request to join the SAFINOUS-CABG Consortium and share published and/or unpublished IPD from their center. If we did not receive a response after 2 weeks, the authors were contacted again. From the 48 eligible studies (Table E1),^3^, ^4^, ^5^, ^6^, ^7^^,^^14^, ^15^, ^16^, ^17^, ^18^, ^19^, ^20^, ^21^, ^22^, ^23^, ^24^, ^25^, ^26^, ^27^, ^28^, ^29^, ^30^, ^31^, ^32^, ^33^, ^34^, ^35^, ^36^, ^37^, ^38^, ^39^, ^40^, ^41^, ^42^, ^43^, ^44^, ^45^, ^46^, ^47^, ^48^, ^49^, ^50^, ^51^, ^52^, ^53^, ^54^, ^55^, ^56^ we received a response from 8 institutions, and a final of 5 centers that used uniform surgical revascularization strategies were included in the IPD meta-analysis (Table E2). For left anterior descending revascularization, a LIMA graft was used and additional SVGs in the case of significant diagonal disease. The use of composite (ie, SVG Y-grafts or LIMA/SVG grafts) or sequential grafts was overall minimal (∼10%) and decided by the operating surgeon. Patients routinely received statin and antiplatelet treatment postoperatively as per standard local clinical practice. Participant demographics, patient-related risk factors, procedural details, outcome, and follow-up data were extracted from the received IPD files and aggregated into a database after careful data examination.

### Statistical Analysis

#### Arm A: Aggregate data meta-analysis

The meta-analysis of the reported proportions of graft occlusion in eligible studies was carried out using a random effects model using the method of DerSimonian and Laird,^57^ with the estimate of heterogeneity being taken from the inverse-variance random-effect model (metaprop command; Stata Statistical Software, Release 13; StataCorp LP, College Station, Tex). Subgroup and meta-regression analysis were carried out to identify predictors of reported graft occlusion rates (metareg command; Stata). To explore the association between the period of patient enrollment and graft occlusion in meta-regression, a “chronological rank” was assigned to all studies. A random-effects model was used to obtain the pooled incidence of SVG occlusion (and 95% confidence intervals [CIs]) and illustrated in forest plots. Subgroup analyses were performed for the time of graft patency assessment postsurgery (<1 month, 1 to <3 months, 3 to 6 months, or 12 months), the type of surgery performed (on-pump vs off-pump surgery), period of patient enrollment, study location, and study size. The presence of statistical heterogeneity was explored using the I^2^.

#### Arm B: IPD predictive model development

The shared IPD contributed to the formation of a database of 1864 patients (2925 SVGs) with complete angiographic follow-up data on early SVG occlusion. The collected demographic characteristics were examined for the extent of missingness. Missing values occurred for several predictors in our dataset, and some variables were systematically missing, meaning they were not collected within specific studies. We therefore applied a multilevel multiple imputation (MLMI) model, which uses generalized linear mixed effect model to simultaneously impute sporadically and systematically missing variables in the setting of IPD meta-analysis. MLMI also fully accounts for between-study heterogeneity within the imputation model.^58^ Simulation studies have shown that MLMI is associated with less bias in predictor effects compared with a complete case analysis—where studies with systematically missing variables are excluded—and MLMI is also associated with less bias than traditional multiple imputation, which ignores heterogeneity across studies.^58^ All variables that were available in at least 70% of participants across all studies combined were considered for inclusion in the multiple imputation model. Five imputation data sets were generated.

The population of the SAFINOUS-CABG Consortium was split using a random seed into a derivation (80%, n = 1492 patients) and validation (20%, n = 372 patients) cohort for prediction of SVG occlusion (caret package, R project). The derivation cohort was used for model development and internal 10-fold cross-validation (and an optimism-adjusted c-index was also calculated), whereas the validation cohort served for the validation of the developed model. All variables included in the imputation model were included in a generalized logistic random effects model (lme4 R package) as predictors for graft occlusion within the 1st year post surgery (using a random effect for individual cohorts, ie, surgical sites). A random effects model assumes that patient level observations are not independent as in the case of samples drawn from multiple sites. All remaining predictor variables were introduced as fixed effects. The model included demographic variables (age, sex, body mass index), cardiovascular risk factors (hypertension, dyslipidemia, diabetes mellitus, smoking), clinical scores (ie, New York Heart Association class), laboratory/diagnostic (preoperative serum creatinine levels, left ventricular ejection fraction, number of diseased vessels), and procedural characteristics (endoscopic vein harvesting, on/off-pump operation, use of complex, ie, composite or sequential, grafts, number of grafts, and target vessel type). All continuous predictors were included as linear terms in the regression model because this was found to be a good approximation based on assessment for nonlinearity using fractional polynomials. The discriminatory performance of prediction models was assessed using the c-index. Model calibration was assessed graphically using a calibration plot and a smoothed loess estimator.

The final model was used for the construction of the SAFINOUS risk score by following the method described by Sullivan and colleagues^59^ previously used in the development of the Framingham risk score system. To summarize in brief, points were assigned to each variable using as 1 point the risk related with a 10-year increment in age (constant B = 10 × 0.015 = 0.15) and rounded to the nearest integer. Continuous variables were categorized, and each category was assigned point scores based on the distance of each category from the reference one. Points were assigned to each variable by considering the beta coefficients of the final model. The performance of the model across patient subgroups was explored using ipdover and ipdmetan commands in Stata All analyses were completed with R (www.r-project.org; version 3.2.4) and Stata version 13.0 (StataCorp LP).

## Results

### Incidence of Early SVG Occlusion in Published Literature

**Figure 2:**
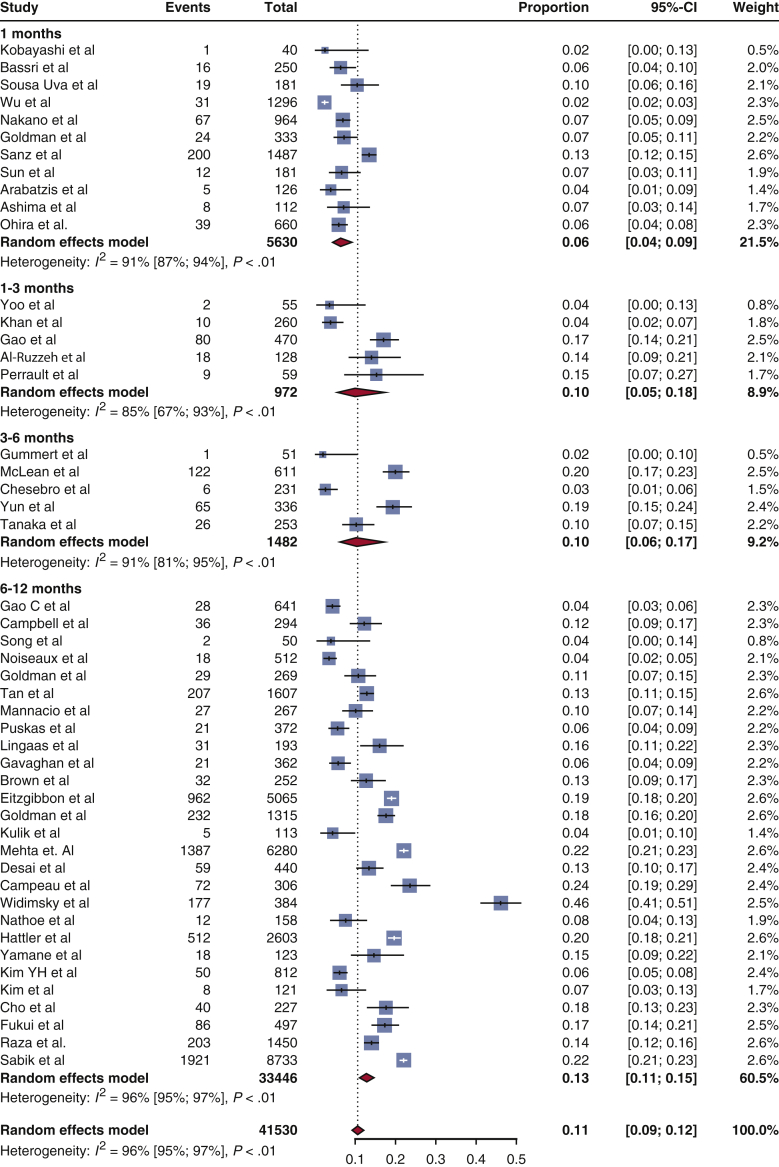
Early saphenous vein graft occlusion in published literature. Forest plot of published studies for the incidence of saphenous vein graft occlusion during the first 12 months post-coronary artery bypass grafting. The size of the squares corresponds to the weight of each study. The *diamonds* and their *width* represent the pooled weighted effect size and the 95% confidence intervals (*CI*), respectively.

**Figure 3:**
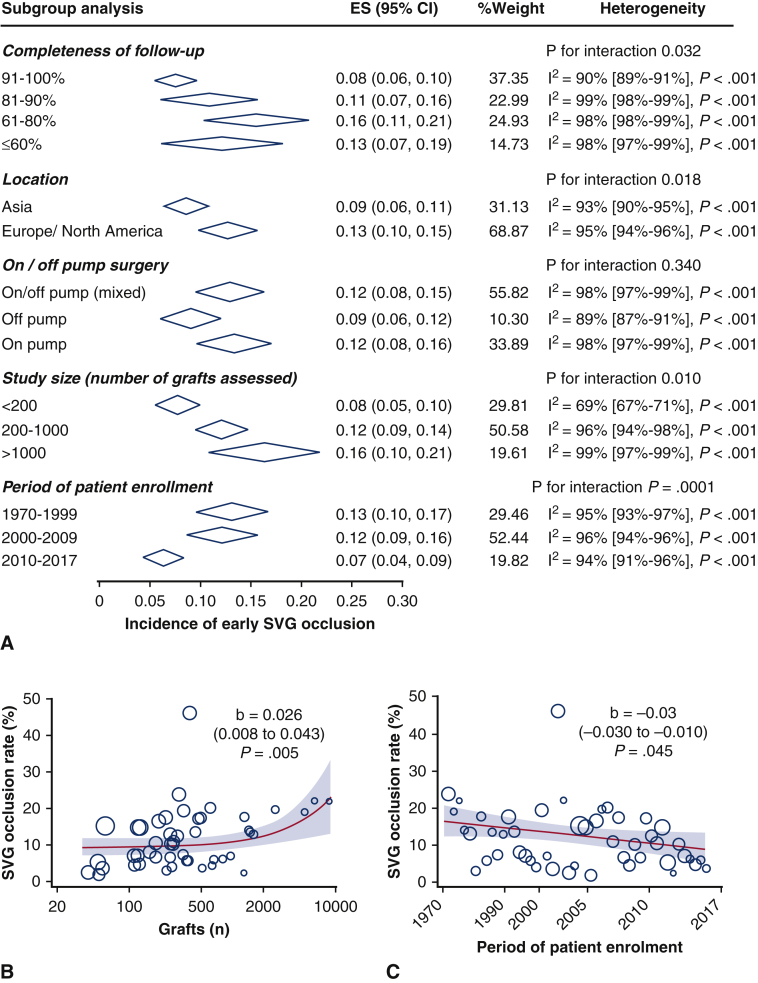
Subgroup analysis for the aggregate data meta-analysis. Subgroup analysis for completeness of angiographic follow-up, study location, on/off-pump surgery, study size, and period of patient enrollment (A). Meta-regression and bubble plots for the association between the incidence of graft occlusion and study size (number of grafts assessed, B) or the period of patient enrollment (chronological ranking of studies, C). The size of the *circles* represents the weight of each study on the pooled estimate for graft occlusion. *ES*, Effect size; *CI*, confidence interval; *SVG*, saphenous vein graft.

Among 10,332 abstracts identified with our literature search strategy, a total of 48 clinical studies met the eligibility criteria. The incidence of SVG occlusion and the detailed characteristics of the identified studies^3^, ^4^, ^5^, ^6^, ^7^^,^^14^, ^15^, ^16^, ^17^, ^18^, ^19^, ^20^, ^21^, ^22^, ^23^, ^24^, ^25^, ^26^, ^27^, ^28^, ^29^, ^30^, ^31^, ^32^, ^33^, ^34^, ^35^, ^36^, ^37^, ^38^, ^39^, ^40^, ^41^, ^42^, ^43^, ^44^, ^45^, ^46^, ^47^, ^48^, ^49^, ^50^, ^51^, ^52^, ^53^, ^54^, ^55^, ^56^ are presented in Table E1. In the meta-analysis of aggregate published data (Figure 2), the pooled estimate for SVG occlusion rate was 6% of grafts at 1 month (data on 13,944 grafts) and 13% of grafts at 12 months (data on 33,446 grafts). The estimated risk of SVG occlusion within the first year post-CABG was 11% of grafts (data on 41,530 grafts). There was significant heterogeneity between studies (I^2^ = 98%, *P* < .001), which was partly explained by differences in the completeness of angiographic follow-up, study location, study size, and period of patient enrollment (Figure 3, *A*). In more recent studies (period of patient enrolment after 2010), the incidence of early SVG occlusion was estimated at 7%. Similar results were identified in meta-regression analysis, where there was a significant association of graft occlusion with larger and older studies (Figure 3, *B* and *C*) cohorts in the field. In multivariate meta-regression analysis, the size of the study, the date of the cohort, and the time point of graft patency assessment post-CABG could explain 16.4% of between-study variance.

### IPD Meta-Analysis and Prediction Modeling for Early SVG Occlusion

**Figure 4:**
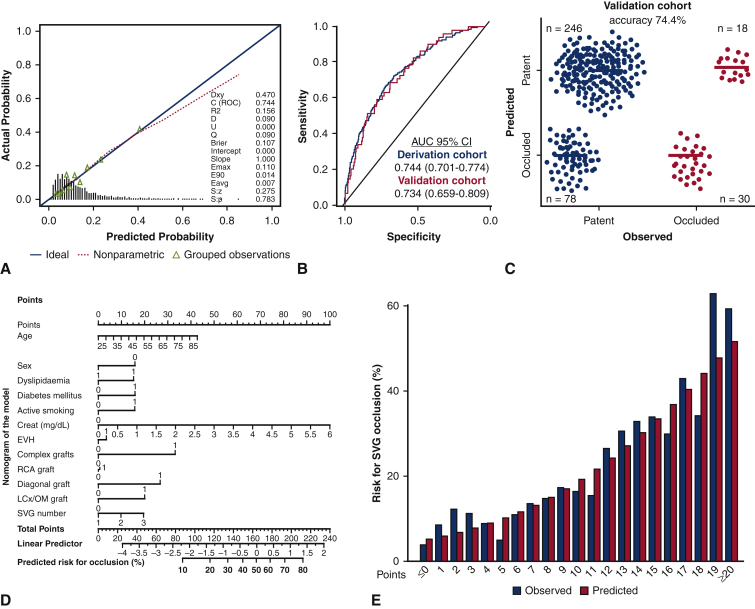
Development of a predictive model for early saphenous vein graft (*SVG*) occlusion. Calibration curve and goodness of fit for the developed prediction model for SVG occlusion (A) and area under the curve (*AUC*) for model's performance in derivation and validation cohorts (B). Confusion matrix for predicted versus observed SVG occlusion in the validation cohort based on the developed model (C). Nomogram for predicting the risk for early SVG occlusion from the developed model (D). Observed and predicted probabilities for SVG occlusion across strata of the constructed SAFINOUS score, derived from the final predictive model (E). *CI*, Confidence interval; *EVH*, endoscopic vein harvesting; *RCA*, right coronary artery; *LCx*, left circumflex artery; *OM*, obtuse marginal; *SVG*, saphenous vein graft.

**Figure E2:**
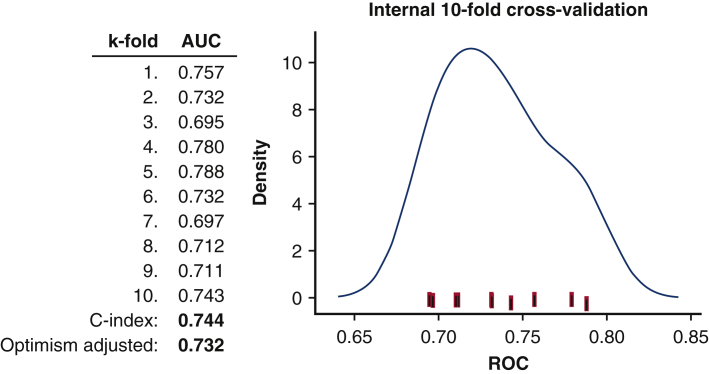
Model training with internal validation in the derivation cohort with 10-fold cross-validation and optimism adjusted c-index. *AUC*, Area under the curve; *ROC*, receiver operating characteristic.

For the SAFINOUS-CABG Consortium IPD, the detailed demographic characteristics of the population included in the derivation and validation cohorts are summarized in Table 1 (the individual cohorts contributing to the shared IPD are shown in Table E2). In multivariate analysis age, sex, body mass index, dyslipidemia, diabetes mellitus, active smoking, preoperative creatinine levels, on-pump surgery, endoscopic vein harvesting, use of complex grafts, target vessel type, and SVG number were identified as independent predictors for SVG occlusion during the first year post-CABG (Table 2). The final constructed model had good performance for prediction of early SVG occlusion in the derivation cohort (c-index = 0.744; 95% CI, 0.701-0.774). In 10-fold cross-validation (Figure E2) the optimism-adjusted c-statistics was consistent (adjusted c-index: 0.732). In the validation cohort the model also had good performance (c-index = 0.734; 95% CI, 0.7659-0.801, Figure 4, *B*), and a predictive accuracy of 74.4% for SVG occlusion based on the optimal cut-off (Figure 4, *C*). A nomogram for predicting early SVG occlusion from the developed full clinical model is presented in Figure 4, *D*.

**Table 1:** Study population demographics

	Derivation cohort	Validation cohort
	(n = 1492)	(n = 372)
Age, y	61.48 (8.89)	60.83 (9.41)
Male sex, %	78.9	82.0
Body mass index, kg/m^2^	27.25 (4.17)	27.49 (4.49)
Hypertension, %	62.7	62.4
Dyslipidemia, %	69.2	67.7
Diabetes, %	22.6	22.3
Active smoking, %	25.8	22.3
NYHA class, %		
I	48.5	46.8
II	37.2	39.7
III	10.9	10.5
IV	3.4	3.0
LVEF <50%, %	30.8	31.5
Number of VD, %		
1	1.2	1.3
2	18.8	18.9
3	80.0	79.8
Creatinine, mg/dL	1.03 (0.33)	1.03 (0.36)
Endoscopic harvesting, %	20.7	22.2
On pump, %	45.8	45.7
Number of SVGs, %		
1	53.8	55.1
2	37.2	37.4
3	7.5	6.2
≥4	1.5	1.4
Complex SVGs, %	9.8	10.4
Target vessel diagonal, %	28.0	27.5
LCx/OM, %	52.8	52.1
RCA, %	74.1	76.6
Occlusion (% patients)	13.7	13.7

Continuous variables presented as mean ± standard deviation. *NYHA*, New York Heart Association; *LVEF*, left ventricular ejection fraction; *VD*, vessel disease; *SVG*, saphenous vein graft; *LCx*, left circumflex; *OM*, obtuse marginal; *RCA*, right coronary artery.

**Table 2:** Independent predictors for early saphenous vein graft occlusion in the final model

Characteristics	Coding	Regression coefficients	Odds ratio (95% CI)
Age, y	–	0.015	1.02 (1.01-1.02)
Sex	Male	–0.632	0.53 (0.46-0.62)
Dyslipidemia	–	0.284	1.33 (1.14-1.55)
Diabetes mellitus	–	0.361	1.43 (1.25-1.65)
Active smoking	–	0.452	1.57 (1.35-1.82)
Creatinine, mg/dL	–	0.546	1.73 (1.46-2.04)
Harvesting technique	EVH	–0.426	0.65 (0.51-0.83)
Complex grafts	–	0.632	1.88 (1.58-2.24)
Graft in diagonal	–	0.643	1.90 (1.57-2.30)
Graft in LCx/OM	–	0.615	1.85 (1.50-2.28)
Graft in RCA	–	0.386	1.47 (1.22-1.78)
Number of SVG	–	0.291	1.34 (1.12-1.60)
Intercept		–4.58	

*CI*, Confidence interval; *EVH*, endoscopic vein harvesting; *LCx*, left circumflex artery; *OM*, obtuse marginal; *RCA*, right coronary artery; *SVG*, saphenous vein graft.

### SAFINOUS Score: A Novel Risk Score for Early SVG Occlusion

**Figure 5:**
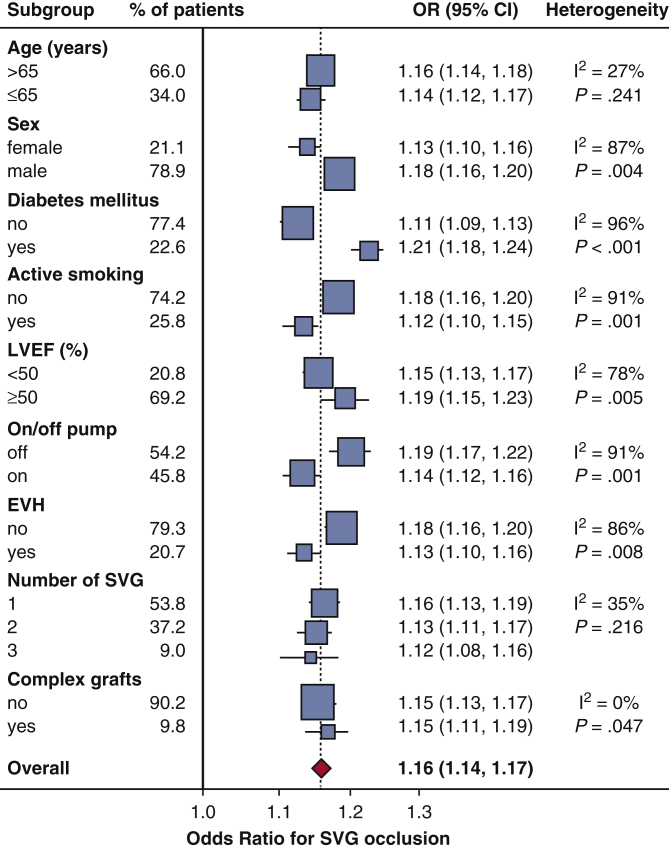
Predictive value of SAFINOUS score for early saphenous vein graft occlusion across patient subgroups. *OR*, Odds ratio; *CI*, confidence interval; *LVEF*, left ventricular ejection fraction; *EVH*, endoscopic vein harvesting; *SVG*, saphenous vein graft.

Based on the final model for SVG occlusion (Table 2), risk score points were assigned to each predictor (Table 3), to construct a risk score system. The SAFINOUS score comprised 12 risk variables: (1) demographic characteristics (age, sex), (2) clinical risk factors (dyslipidemia, diabetes, active smoking), (3) laboratory findings (preoperative creatinine levels), and (4) operative characteristics (endoscopic vein harvesting, use of complex grafts, target vessel types [graft in diagonal, graft in left circumflex, graft in right coronary artery], and number of SVGs). The SAFINOUS risk score performed similarly well compared with the original model for prediction of SVG occlusion (c-index = 0.700; 95% CI, 0.684-0.716). For a classification cut-off of 0.25 in logistic regression, the SAFINOUS score correctly classified 83.7% of patients assessed (sensitivity 27%, specificity 93%, positive predictive value 37%, negative predictive value 90%). The predicted and observed probabilities across different points of the SAFINOUS score are presented in Figure 4, *E*. In subgroup analysis SAFINOUS score retained its predictive value for SVG occlusion across all patient subgroups (Figure 5).

**Table 3:** Predictors from logistic regression used in the construction of the SAFINOUS score

Variable	Categories	Reference	W_ij_	β_i_	β_i_ (W_ij_ –W_iref_)	Points
Age, y	30-39	34.5	W_1ref_	0.015	–0.30	–2
	40-49	44.5			–0.15	–1
	50-59	54.5			0	0
	60-69	54.5			0.15	1
	70-79	74.5			0.30	2
	80-89	84.5			0.45	3
Sex		Male	W_2ref_	–0.632	–0.632	–3
Dyslipidemia			W_4ref_	0.284	0.284	1
Diabetes			W_5ref_	0.361	0.361	3
Active smoking			W_6ref_	0.452	0.452	3
Creatinine, mg/dL	0.50-0.99	0.75	W_7ref_	0.546	–0.273	–2
	1.00-1.49	1.25			0	0
	1.50-1.99	1.75			0.273	2
	2.00-2.49	2.25			0.546	4
	≥2.50	3.50			1.229	8
Endoscopic harvesting			W_9ref_	–0.426	–0.426	–3
Complex grafts			W_10ref_	0.632	0.632	4
Number of SVG	1	1	W_11ref_	0.291	0	0
	2	2			0.291	2
	≥3	3			0.582	4
Graft in diagonal			W_12ref_	0.643	0.643	4
Graft in LCx/OM			W_13ref_	0.615	0.615	4
Graft in RCA			W_14ref_	0.386	0.386	3

Points were assigned to each variable using as 1 point the risk related with a 10-year increment in age (constant B = 10 × 0.015 = 0.15) and rounded to nearest integer. *SVG*, Saphenous vein graft; *LCx*, left circumflex artery; *OM*, obtuse marginal; *RCA*, right coronary artery.

## Discussion

This study provides a comprehensive prediction model for early SVG occlusion. We first showed that based on aggregate data from 48 studies, the pooled incidence of SVG occlusion within the first year post-CABG is estimated at 11%, whereas it is significantly lower with modern surgical practice techniques, estimated at 7%—in studies with period of patient enrollment after 2010. Then by using IPD, we developed and validated a predictive model, which was used to construct the SAFINOUS score, a 12-variable risk score point system, for the calculation of the individualized risk for early SVG occlusion in patients undergoing CABG. Taken together, our findings (Figure 1) could contribute to the risk stratification of patients for early SVG occlusion, guide operation planning as well as the postoperative patient management and the deployment of tailored, preventive therapeutic strategies (Video 1).

**Video 1:**
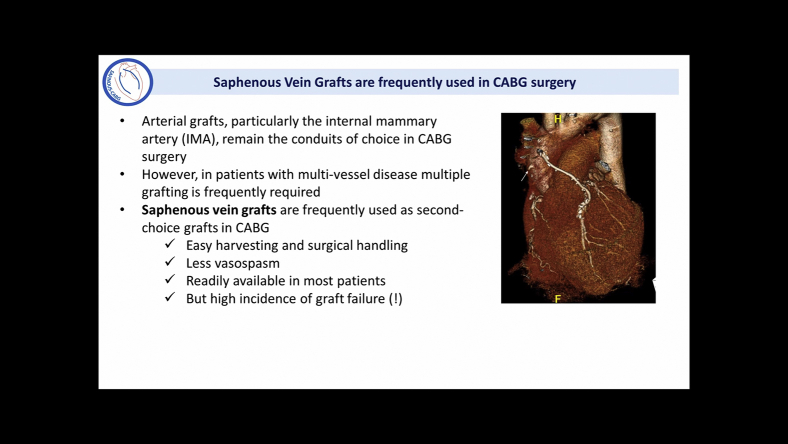
The main findings and implications of the study are summarized in this online video by Dr Antonopoulos. Video available at: https://www.jtcvs.org/article/S0022-5223(19)31640-X/fulltext.

CABG is the gold-standard revascularization strategy for patients with complex multivessel coronary artery disease, since it reduces mortality and major adverse cardiac events and improves the quality of life of patients.^60^ Nevertheless, the efficacy of CABG is hampered by the high occlusion rates of SVGs, estimated as high as 43% at 10 years. SVG occlusion has a negative impact on the quality of life of patients^61^ and poses an extra economic burden to health care systems. SVG occlusion leads to recurrent angina symptoms, heart failure development, and significantly poorer clinical outcomes in the setting of acute myocardial infarction compared with native vessel disease.^62^ Revascularization of occluded SVGs is also challenging and has a high rate of complications.^62^

In contrast to late SVG occlusion (which is due to atherosclerosis development) early SVG occlusion is attributed to graft thrombosis or accelerated intimal hyperplasia, developed rapidly when the SVG is exposed to arterial circulation.^1^ Although prediction models have been constructed for late graft disease (ie, SVG atherosclerosis),^4^ the factors associated with the risk of early SVG occlusion have not been systematically evaluated, and a widely accepted comprehensive prediction model for early SVG occlusion had been lacking. We have now developed and validated a prediction model for graft occlusion within the first-year post-CABG by using multicenter IPD. We have identified clinical, technical, and perioperative predictors for early SVG occlusion. Female sex is independently associated with early SVG occlusion, as previously shown,^6^ possibly due to the smaller vessel size of female subjects. Cardiovascular risk factors, eg, diabetes, dyslipidemia, smoking, and chronic kidney disease^63^ also raise the risk for early SVG occlusion. The use of complex grafts, grafting of secondary coronary branches, and the harvesting technique were also independent risk factors for graft occlusion. Although previous reports suggest greater rates of graft failure with endoscopic vein harvesting,^64^ within the SAFINOUS-CABG IPD Consortium endoscopic vein harvesting was associated with reduced risk for occlusion. This could reflect the experienced harvesters employed in the centers of the Consortium.

The presented SAFINOUS score is the first comprehensive attempt to develop a prediction model for early graft occlusion that could be used for the risk stratification of patients undergoing CABG. The proposed SAFINOUS score could be used as a clinical decision-making tool to estimate the personalized risk for early SVG occlusion. Assessment of SAFINOUS score could help in surgery planning preoperatively (eg, total arterial revascularization) or the tailored administration of aggressive treatment postoperatively (eg, dual antiplatelet therapy) as a more cost-effective strategy to reduce cardiovascular events and bleeding complications.

Certain limitations of our study should be acknowledged. First, the developed model may not account for possible residual confounding or unchecked interactions and is subject to selection bias (typical for observational studies). The absence of data on storage solutions and grafting of small target vessels are also weaknesses of the developed model. Future iterations of the model in a larger cohort with available biological data on harvested SVGs could be used to evaluate the incremental value of biological factors for prediction of graft failure on top of the constructed SAFINOUS score. Finally, although a rigorous statistical approach was used to develop the model, including internal and external validation, stronger forms of validation may be required. For example, future validation studies from diverse geographic regions, with different population background and operation strategies, as well as full independent validation by independent investigators would be welcome.

## Conclusions

We conducted a systematic review of the published literature for the incidence of early SVG occlusion and present the first comprehensive prediction model for early SVG occlusion based on a large multicenter cohort of patients undergoing CABG. SAFINOUS score is a 12-variable risk score point system that independently predicts early SVG occlusion across all patient subgroups and could be used in clinical practice to identify high-risk individuals for reduced graft patency. SAFINOUS score could contribute to the design of clinical studies to test the effectiveness of tailored dual antiplatelet and/or aggressive lipid lowering treatment in patients at high risk for early graft occlusion or the efficacy of novel therapeutic strategies.

### Conflict of Interest Statement

Authors have nothing to disclose with regard to commercial support.

## Figures and Tables

**Table E1:** Summary of identified studies on early saphenous vein graft patency post-coronary artery bypass operation

Study	Period	Size, n	Grafts, n	On/off pump	Time point	Modality	Completeness FU, %	Occlusion (%)			
								0-1 m	3 m	6 m	12 m
Sabik et al^47^	1972-1999	4333	8733	On/off	1 y	ICA	9	–	–	–	22.0
Mehta et al^39^	2002-2003	3,014	6,280	On/off	<1 y	ICA	79	–	–	–	22.1
Nakano et al^40^	2000-2005	1422	964	Off	<1 mo	ICA	89	7.0	–	–	–
Fitzgibbon al^3^	1969-1994	1388	5,065	On	<1 mo and 1 y	ICA	83	12.0	–	–	19.0
Hattler et al^31^	2002-2007	1370	2,603	On/off	<1 y	ICA	62	–	–	–	19.7
Ohira et al^43^	<2017	1176	660	On/off	1 wk	CTA	35	6.0	–	–	–
Raza et al^4^	1972-2011	985	1450	On/off	1 y	ICA	21	–	–	–	14.0
Fukui et al^23^	2004-2009	930	497	Off	<1 mo and 1 y	ICA	48	–	–	–	17.4
Tan et al^7^	1987-1990	912	1607	On	1 y	ICA	96	–	–	–	12.9
Sanz et al^48^	1984-1988	741∗	1487	On	<1 mo	ICA	83	13.5	–	–	–
Wu et al^53^	2009-2012	582	1296	On	<1 mo	ICA	90	2.4	–	–	–
Desai et al^22^	1996-2001	440	440	On	12 mo	ICA	78	–	–	–	13.6
Goldman al^27^	1983-1986	406∗	1,315	On	1 wk and 1 y	ICA	81	–	–	–	17.7
Kim YH et al^34^	2011-2014	368	812	Off	1 wk and 1 y	ICA	60	2.0	–	–	6.2
Goldman et al^29^	2003-2009	367	269	On/off	1 wk and 12 mo	ICA	97	3.0	–	–	11.0
McLean et al^6^	2003-2006	291	611	On/off	<6 mo	CTA	81	–	–	20.1	–
Widimsky et al^5^	<2004	255	384	On/off	1 y	ICA	64	–	–	–	46.2
Kim KB et al^33^	1998-2001	240	121	Off	<1 mo and 1 y	ICA	60	2.4	–	–	7.1
Gao C et al^24^	2002-2006	237	641	On/off	1 mo and 1 y	CTA	100	1.1	–	–	4.4
Gao G et al^25^	2007-2008	224	470	On/off	3 mo	CTA	90	–	17.2	–	–
Tanaka et al^52^	2009-2012	199	253	On/off	<6 mo	ICA	64	–	–	10.3	–
Puskas et al^46^	2000-2001	197	372	On/off	1 mo and 1 y	ICA	93	2.3	–	–	5.8
Oshima et al^44^	2009-2015	196	112	On/off	1 mo	ICA	40	7.1	–	–	–
Goldman et al^28^	1977-1981	176∗	333	On	1 wk	ICA	72	7.4	–	–	–
Chesebro et al^20^	1986-1988	176∗	231	On	<6 mo	ICA	88	–	–	3.0	–
Campeau et al^19^	1969-1972	172	306	On	2 wk and 1 y	ICA	70	11.0	–	–	23.8
Kobayashi et al^35^	2002-2004	167	40	On/off	<1 mo	ICA	100	2.5	–	–	–
Noiseaux et al^42^	<2017	157	512	On/off	1 y	CTA	98	–	–	–	3.7
Al-Ruzzeh et al^14^	<2006	151	128	On/off	3 mo	ICA	90	–	14.8	–	–
Sousa Uva et al^50^	<2010	147	181	On/off	1 mo	CTA	94	10.5	–	–	–
Mannacio et al^38^	2006-2009	144	267	Off	12 mo	CTA	96	–	–	–	10.2
Yun et al^56^	2000-2002	144	336	On	6m	ICA	72	–	–	19.4	–
Campbell et al^18^	<2009	130	294	On	<1 y	CTA	100	–	–	–	12.5
Gavaghan et al^26^	1984-1987	127∗	362	On	1 wk and 1 y	ICA	92	1.6	–	–	5.8
Yamane et al^54^	2007-2014	123	123	On/off	1 wk	CTA	62	4.5	–	–	14.8
Lingaas et al^37^	<2006	120	193	On/off	0.3 and 12 mo	ICA	93	3.0	12.5	–	16.5
Kulik et al^36^	2006-2009	113	n/a	On/off	1 y	ICA	81	–	–	–	4.5
Cho et al^21^	1995-1997	109	227	On	1 y	ICA	55	–	–	–	17.6
Bassri et al^16^	2007-2008	107	250	On/off	<1 mo	CTA	100	6.6	–	–	–
Khan et al^32^	2000-2002	104	260	On/off	3 mo	ICA	99	–	4.0	–	–
Brown et al^17^	1976-1980	83∗	252	On	1 y	ICA	86	–	–	–	13
Sun et al^51^	2006-2008	79	181	On/off	1 mo	CTA	80	6.7	–	–	–
Arampatzis et al^15^	<2016	75	126	On/off	1 wk	CTA	50	4.7	–	–	–
Nathoe et al^41^	1998-2000	70	158	On/off	1 y	ICA	64	–	–	–	8.0
Gummert et al^30^	<2006	54	51	On/off	6 mo	CTA/ICA	94	–	–	1.9	–
Yoo et al^55^	<2002	42	55	On/off	<3 mo	CTA/ICA	100	–	3.6	–	–
Perrault et al^45^	2004	35	59	On/off	3 mo	ICA	80	–	15.2	–	–
Song et al^49^	2008-2009	25	50	Off	1 wk and 1 y	CTA	100	0.0	–	–	5.3
Total		23,103	41,530	Number of SVGs assessed		13,944	1165	1482	33,446		

*FU*, Follow-up; *on*, on-pump coronary artery bypass; *off*, off-pump coronary artery bypass; *ICA*, invasive coronary angiography; *CTA*, computed tomography angiography; *n/a*, not available.

∗ Placebo group not included in summary statistics (study numbers correspond to the reference list of the main manuscript).

**Table E2:** Demographic characteristics of clinical cohorts included in the IPD analysis

	Oxford, United Kingdom	Baltimore, Md	Montreal, Canada	Naples, Italy	Toronto, Canada	Total
Study population, n	226	295	35	868	440	1864
Clinical demographics						
Age, y	65.5 (9.3)	63.4 (10.0)	66.2 (9.3)	59.7 (8.2)	60.8 (8.4)	61.3 (9.0)
Males, %	86.7	79.1	97.2	73.0	87.0	79.5
BMI, kg/m^2^	28.1 ± 4.3	29.8 ± 6.2	28.2 ± 3.6	26.5 ± 3.2	–	27.3 (4.2)
Hypertension, %	74.3	82.1	86.1	60.6	46.1	62.7
Dyslipidemia, %	89.8	83.7	94.4	57.7	68.5	68.9
Diabetes, %	23.9	34.8	27.8	15.9	26.1	22.5
Previous MI, %	47.8	40.5	–	–	46.1	48.7
NYHA class, %						
I/II	44.9/43.1	88.5/3.4	32.8/50.0	38.4/47.1		48.0/37.8
III/IV	10.2/1.8	5.4/2.7	11.8/5.9	11.1/3.5	–	10.7/3.5
LVEF <50%, %	14.3	0.3	20.6	35.7	51.4	31.0
Creatinine, mg/dL	0.93 (0.21)	1.00 (0.24)	–	1.05 (0.43)	1.05 (0.210)	1.03 (0.34)
Number of VD, %						
1	4.9	1.4	0.0	0.0		1.1
2	22.1	14.9	14.3	22.9		19.0
3	73.0	83.7	85.7	77.1		79.9
Operative characteristics, %						
Urgent	0.0	37.6	0.0	–	33.0	23.9
On-pump	18.6	97.6	88.9	5.9	100	45.8
EVH	86.3	53.4	–	0.0	0.0	21.0
SVG number, %						
1	37.2	26.4	80.0	43.4	100.0	54.0
2	48.2	42.4	20.0	52.2	0.0	37.2
≥3	14.6	31.2	0.0	4.4	0.0	8.8
Target vessel, %						
Diagonal	40.3	50.9	17.1	15.0	–	27.8
LCx/OM	61.1	69.2	22.9	52.9	–	52.6
RCA	69.9	66.1	60.0	94.6	–	74/8
Complex grafts, %	3.2	–	2.9	2.9	–	9.3
Follow-up time, mo	<12	6	3	12	12	<12
Occlusion, %	11.5	33.4	8.5	7.6	13.6	13.7

*BMI*, Body mass index; *MI*, myocardial infarction; *NYHA*, New York Heart Association; *LVEF*, left ventricular ejection fraction; *VD*, vessel disease; *EVH*, endoscopic vein harvesting; *SVG*, saphenous vein graft; *LCx*, left circumflex; *OM*, obtuse marginal; *RCA*, right coronary artery.

## Supplementary Data

Video 1 The main findings and implications of the study are summarized in this online video by Dr Antonopoulos. Video available at: https://www.jtcvs.org/article/S0022-5223(19)31640-X/fulltext.

TRIPOD Checklist
